# Death of Professor Drake

**Published:** 1853-01

**Authors:** 


DEATH OF PROFESSOR DRAKE.

The Medical Profession has been thrown into a state of mourning by the recent decease of this eminent and distinguished Physician and Lecturer. He died in this city, 5th of November, after a few days illness, commencing with an attack of Influenza and terminating in cerebral congestion. At the time of his decease he held the Chair of Practice in the Medical College of Ohio. As a lecturer, his reputation has been in the ascendant for more than twenty years. The tokens of respect bestowed have been with us universal, all the Medical bodies of the city, Covington, Newport &c., joined in the funeral obsequies. As a contributor to medical science, Dr. Drake has but few equals, and it is universally regretted that he should have been cut off before his work on the diseases of the Great Mississippi Valley was completed.

To Dentistry, as a science, Dr. Drake lent his hearty co-operation, regarding with much satisfaction the efforts now being made to advance this speciality of Medical and Surgical science. Of his interest in this we have repeated evidence. Of him we can truly say " the dead live in their works."

